# How should a general practitioner say no to a patient?

**DOI:** 10.1080/02813432.2023.2250398

**Published:** 2023-08-28

**Authors:** Mikael O. Ekblad

**Affiliations:** Specialist in General Practice, Department of General Practice, University of Turku and Turku University Hospital, Turku, Finland

The authority of general practitioners (GPs) in relation to their patients has undergone a substantial weakening over the past few decades. The once stringent hierarchical dynamic has evolved into a more relaxed and equal discourse. However, this shift has also resulted in the loss of the ability to give people vigorous instructions. Nowadays, communication skills are almost as vital as clinical expertise in a GP's profession. It is necessary to be able to justify the background of the decisions for the patients more widely than before, especially if the decision differs from the patient's expectations. A Norwegian study including 707 patients showed that good communication skills of the GP was most frequently rated as “very important” when assessing the importance of different aspects of general practice (1). It has become more and more common for the patients to rate the GP’s appointments afterwards. The rating is likely to emphasize the interaction between GP and patient, not necessarily the quality of care or the GP's skills. The patient-consumer paradigm has become more pervasive in healthcare settings.

Patients most commonly visit their GP due to uncertainty surrounding their health concerns. Patients seek for information about their own symptoms on the internet, and often before the appointment they already have an idea and wishes about what should be examined. This trend has also led to patients expressing explicit requests for specific tests and medications, while concurrently declining certain treatments or independently discontinuing medications. It is especially challenging when the patient's and GP's views on the need for further examinations do not meet.

At my former workplace, a colleague who had previously been working in hospital sector came to work in primary health care center for a few months. This colleague encountered difficulties when declining unnecessary tests requested by patients. When discussing this matter, the colleague stated a preference for being a good doctor rather than solely an empathetic one. However, it's crucial to note that these things are not mutually exclusive. Thus, we discussed this and how to deal with a patient who wants examinations for which the GP does not see an indication. A recent qualitative study investigated GPs' communication strategies to avoid unnecessary medical imaging and patients' experiences with such strategies (2). Five strategies were identified in the study: 1) wait and see – or suggest an alternative; 2) the art of rejection; 3) seek support from a professional authority; 4) partnership and shared decision-making, and 5) reassurance, normalization, and recognition. Interestingly, the study found that GPs often combine multiple strategies to address patient expectations for diagnostic imaging. Notably, patients reported satisfaction regardless of the specific strategy employed by GPs, including instances where the referral request was declined.

GPs frequently encounter patients with medically unexplained symptoms (MUS), which can be challenging to manage. Often the GP understands that it would be important to stop the cycle of unnecessary examinations, but this may lead to challenging situations with the patient. Houwen et al. (3) investigated which are the most important learnable communication elements during MUS consultations according to MUS patients, GPs, MUS experts, and teachers and to explore how these elements should be taught to GPs and GP trainees. Five elements were identified: 1) thorough somatic and psychosocial exploration, 2) communication with empathy, 3) creating a shared understanding of the problem, 4) providing a tangible explanation, and 5) taking control. The authors presented three teaching methods for these elements: 1) awareness and reflection of GPs about their feelings towards MUS patients, 2) assessment of GPs' individual needs, and 3) training and supervision in daily practice (3).

Nowadays, it is emphasized that the patient feels that he has been heard and confronted, even if the GP and the patient do not always agree on the matter. The concept of shared decision-making has gained prominence, particularly in the care of patients with chronic conditions. Nørgaard et al. (4) investigated shared decision-making in general practice from the perspectives of patients with chronic obstructive pulmonary disease (COPD) or type 2 diabetes (T2DM). A total of 468 patients filled in the 9-item Shared Decision-Making Questionnaire ranging from 0 to 5 (best/highest). The overall mean score for all items was 3.7 and the highest scores were for patients with T2DM and the lowest for patients with both T2DM and COPD.

When making decisions together with the patient, unnecessary examinations can be avoided, and it is possible to facilitate proactive planning of treatment. I hope that we, general practitioners, will continue to have the courage to rely on our clinical expertise and to challenge patients' wishes in a constructive way.

## References

[1] Eide TB, Straand J, Braend AM. Good communication was valued as more important than accessibility according to 707 Nordic primary care patients: a report from the QUALICOPC study. Scand J Prim Health Care. 2021 Sep;39(3):296–304. doi: 10.1080/02813432.2021.1928837. 34041993 PMC8475124

[2] Walderhaug KE, Nyquist MK, Mjølstad BP. GP strategies to avoid imaging overuse. A qualitative study in Norwegian general practice. Scand J Prim Health Care. 2022 Mar;40(1):48–56. doi:10.1080/02813432.2022.2036480. 35188069 PMC9090343

[3] Houwen J, Lucassen PLBJ, Stappers HW, van Spaendonck K, van Duijnhoven A, Olde Hartman TC, Dulmen SV. How to learn skilled communication in primary care MUS consultations: a focus group study. Scand J Prim Health Care. 2021 Mar;39(1):101–110. doi:10.1080/02813432.2021.1882088. 33569982 PMC7971340

[4] Nørgaard B, Titlestad SB, Marcussen M. Shared decision-making in general practice from a patient perspective. A cross-sectional survey. Scand J Prim Health Care. 2022 Jun;40(2):167–172. doi:10.1080/02813432.2022.2069700. 35481437 PMC9397466

